# Lessons From Project Northland: Preventing Alcohol Problems During Adolescence

**Published:** 1998

**Authors:** Carolyn L. Williams, Cheryl L. Perry

**Affiliations:** Carolyn L. Williams, Ph.D., is an associate professor and Cheryl L. Perry, Ph.D., is a professor in the Division of Epidemiology, School of Public Health, University of Minnesota, Minneapolis, Minnesota

**Keywords:** community based prevention, prevention program, prevention strategy, adolescent, AOD availability, attitude toward AOD, prevention of AODR (alcohol and other drug related) problems, underage drinker, socioenvironmental factors, junior high school student, high school student, youth culture, harm reduction policy, literature review

## Abstract

Project Northland, an ongoing community trial aimed at reducing alcohol use and alcohol-related problems among adolescents, is nearing completion. The project combines individual-based strategies to encourage adolescents not to use alcohol with community-based strategies to both reduce alcohol availability and modify community attitudes concerning youth drinking. Project Northland has developed prevention programs and followed the same group of adolescents from sixth grade to high school graduation. This article discusses the rationale for this type of program, elements of the adolescents’ social environment targeted for change, the unique challenges of working with high school students compared with younger adolescents, and areas for future research.

Throughout the history of the United States, public opinion concerning alcohol use has oscillated from toleration to disapproval, and the average annual consumption of alcohol has risen and fallen in accordance with this pattern. Current annual per person alcohol consumption among adults in the United States is only about one-third of what it was in the early 19th century (Musto 1996). Along with other indications, a 15-percent drop in adult alcohol consumption since its most recent peak around 1980 (Musto 1996) suggests that a third era of temperance may be under way. Additional evidence to support this shift in American attitudes toward alcohol includes the reinstatement of age 21 as the legal drinking age in all States by the mid-1980s and ongoing public activism, beginning in the mid-1970s, supporting tougher drunk-driving laws.

Despite overall lower alcohol consumption in the United States, American youth drink more at younger ages. One study found that only 9 percent of respondents born between 1919 and 1929 reported first using alcohol (i.e., “you first had a glass of beer or wine, or a drink of liquor such as whiskey, gin, scotch, etc.”) at age 15 or younger, compared with 33 percent of those surveyed who were born between 1971 and 1975 (Johnson and Gerstein 1998). Many youth drink alcohol regularly. In a 1995 survey, 25 percent of 8th graders, 39 percent of 10th graders, 51 percent of 12th graders, and 68 percent of college students reported drinking at least once during the 30 days prior to being surveyed (Johnston et al. 1996). In addition, the survey results suggest that many young drinkers consume multiple drinks per drinking occasion. Fifteen percent of 8th graders, 24 percent of 10th graders, 30 percent of 12th graders, and 40 percent of college students reported consuming five or more drinks in a row at least once in the 2 weeks before the survey (Johnston et al. 1996).

The widespread and often heavy alcohol use by adolescents are associated with significant morbidity and mortality (e.g., Chassin and DeLucia 1996) that are not confined to the group of more extreme users. In fact, because the general population includes more light and moderate drinkers than heavy drinkers, the former experience more alcohol-related problems as a group, even though as individuals they are at less risk than heavier drinkers (National Institute on Alcohol Abuse and Alcoholism [NIAAA] 1994). This finding has tended to shift the focus of prevention efforts away from the identification of problem drinkers toward the prevention of alcohol use during adolescence (Wagenaar and Perry 1995).

Project Northland is the largest community trial in the United States to focus on the prevention of alcohol use and alcohol-related problems among adolescents. Using the example of Project Northland, this article describes a comprehensive approach that combines individual-based strategies to encourage adolescents not to use alcohol with community-based strategies to both reduce alcohol availability and modify community attitudes regarding underage drinking.

Epidemiologic studies demonstrate the importance of delaying the onset of drinking and reducing alcohol use during adolescence. Data from the 1992 National Longitudinal Alcohol Epidemiologic Survey of 27,616 drinkers and former drinkers found that people who started drinking before age 15 were four times more likely to become alcohol dependent at some point during their lives, compared with those who had initiated drinking at age 20 or older (40 percent versus 10 percent) (Grant and Dawson 1997) (see the Epidemiologic Bulletin, pp. 144–150). Similarly, the number of people who experienced alcohol abuse in their lifetime increased as the age of drinking onset decreased (Grant and Dawson 1997).^1^ An earlier British study that followed a sample of young people from ages 16 to 23 (Ghodsian and Power 1987) found that those youth who drank the most in quantity and frequency at age 16 were the most likely to drink heavily at age 23. More specifically, 16-year-old males who reported drinking the week prior to the survey were nearly four times more likely to report heavy drinking (i.e., more than 50 drinks of alcohol in 1 week) at age 23, compared with those who reported being abstinent at age 16 (15 percent versus 4 percent).

A recent behavioral genetics study suggests that social and environmental factors are more important than genetic influences in delaying drinking until age 16. Rose and colleagues (in press) conducted a population-based study that was able to identify all the twins born in Finland between 1975 and 1979 and to enroll them sequentially in the study when they reached age 16. The study included 2,711 total pairs of twins born over the 5-year period and demonstrated that remaining abstinent from alcohol until age 16 was clearly linked to nongenetic influences. Abstinence rates were influenced by sibling interaction effects, parental drinking patterns, and contextual features of the region. Rose and colleagues suggested that regional features may include variables such as local alcohol sales, ease of underage access to alcohol, and exposure to public drinking or intoxication, issues that have been identified as intervention targets by prevention researchers (e.g., Holder et al. 1997; Wagenaar and Perry 1995).

## Adolescents’ Social Environment: Targets for Comprehensive Prevention

**Figure 1:**
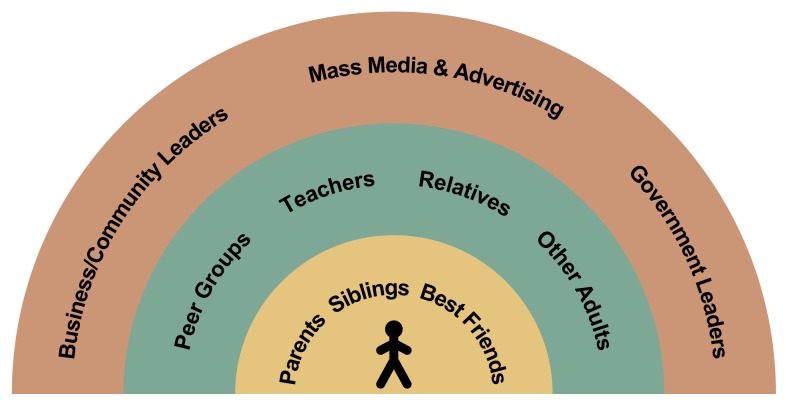
Schematic representation of the social environment of adolescents.

Current prevention efforts tend to be comprehensive and to target factors in the adolescents’ social environment that are known to affect underage drinking (Botvin and Botvin 1992; Dusenbury and Falco 1995; Hansen 1993). Figure 1 illustrates aspects of adolescents’ social environment that can be the targets of prevention efforts.^2^ The most immediate social environment for a given adolescent generally consists of parents, siblings, and best friends. The next ring includes larger peer groups, which may vary by setting (e.g., friends at school, on sports teams, or at religious institutions); teachers; other relatives; and other important adults in an adolescent’s life (e.g., coaches, religious advisors, or other youth group leaders). The outer ring includes the broader community of business (e.g., alcohol merchants, including neighborhood merchants, and major employers in the region) and community leaders as well as local and national government leaders (ranging from school superintendents and police chiefs to mayors and the governor), with the top of the outer ring reserved for mass media and advertising. Project Northland targeted each ring of the adolescents’ social environment.

Much of the popular youth culture—frequently cited by parents, other adults, and youth as among the primary reasons for an adolescent’s use of alcohol, tobacco, and other drugs—is developed primarily in the outermost ring of a young person’s social environment (i.e., mass media, advertising, and businesses that target peer-group identity to encourage youth to engage in various behaviors). For example, advertisements, rock music videos, radio, and youth-oriented publications are adult-created aspects of youth culture that model ways to seek independence and define identity, including sexuality and attractiveness, both of which are crucial developmental issues for adolescents. Popular youth culture influences young peoples’ preferences for clothing, hairstyles, and music within their peer groups. Recent studies document the persuasive and pervasive health-compromising messages in the youth culture promoted by mass media and advertising (e.g., DuRant et al. 1997; Grube and Wallack 1994; Klein et al. 1993; Madden and Grube 1994; Perry 1998). For example, research indicates that sporting events and music videos, which are especially appealing to adolescents, expose youth to extensive alcohol and tobacco use by people they view as positive role models (DuRant et al. 1997; Madden and Grube 1994). In sporting events, beer commercials predominate and include images or themes that portray activities which are dangerous when combined with drinking (e.g., boating).

Until recently, prevention efforts concentrated on changing only family, school, peer, and other immediate interpersonal influences (i.e., the first two rings in figure 1). Even then, interventions with families have been limited (Dusenbury and Falco 1995; Wechsler and Weitzman 1996). Interventions aimed at the outer ring of a young person’s social environment, however, are beginning to show promising results (e.g., Hingson et al. 1996; Holder 1997; Wagenaar et al. in press). A conceptualization of adolescents’ social environment, as shown in figure 1, has guided intervention development for Project Northland (Perry et al. 1996; Williams et al. in press). The project is being conducted in 24 school districts and communities in northeastern Minnesota and is following a cohort of 2,351 adolescents from sixth grade to high school graduation. Project Northland has two distinct phases: early adolescence (Phase 1), which began when the students entered sixth grade (1991) and was completed during their eighth grade school year (1993–1994), and the students’ last 2 years of high school (Phase 2), to be completed in 1999. An interim phase occurred during funding transition, when the students were in the 9th and 10th grades; during that time, they received less intensive interventions.

Project Northland is one of six promising alcohol and other drug prevention programs listed in an independent review as having demonstrated effectiveness (Dusenbury and Falco 1995). The table on page 110 lists the key components of effective prevention programs identified by Dusenbury and Falco and how they are used in Project Northland. Other reviewers have highlighted the same components, which give special emphasis to interactive techniques (e.g., peer leaders or role plays), normative education (e.g., programs to change students’ misconception that underage drinking is more prevalent than it actually is), resistance-skills training (i.e., training to develop skills to resist peer pressure), and broad-based skills training (e.g., leadership skills and skills for identifying unhealthy messages in the media) (e.g., Botvin and Botvin 1992; Hansen 1993; Tobler and Stratton 1997).

The Phase 1 interventions for Project Northland included 3 years of behavioral curricula (Perry et al. 1996; Williams et al. in press), peer leadership (Komro et al. 1996), parental involvement (Williams and Perry 1998; Williams et al. in press), and task forces to initiate community-level changes (Veblen-Mortenson et al. in press). At the end of Phase 1, the intervention group, compared with a control group, demonstrated statistically significant reductions in the onset and prevalence of drinking (i.e., among all students there was a 29-percent reduction in past-week alcohol use and a 19-percent reduction in past-month use) (Perry et al. 1996). The reductions were attributed primarily to changes in peer norms (i.e., young peoples’ views of the acceptability of underage drinking) and peer drinking behavior, parent-child communication that reinforced abstention, increased negative perceptions about the consequences of alcohol use (e.g., “it would hurt my reputation,” “using alcohol could threaten my eligibility to participate in sports,” and “I would be breaking school rules and policies”), and increased resistance skills, according to students’ survey responses (Perry et al. 1996). The greatest program benefits were found for students who had not yet started drinking when the intervention began in 1991 (Perry et al. 1996; Williams et al. in press).

**Table:** Key Components of Effective Prevention Programs^1^ and Their Use in Project Northland

Key Components	Use in Project Northland
Research based/theory driven	Social learning theory was used to develop interventions to decrease alcohol use and related problems among adolescents through strategies to encourage adolescents not to drink, reduce alcohol availability, and modify community attitudes concerning youth drinking.
Developmentally appropriate information about alcohol and other drugs	Early adolescent programs began in sixth grade with education for parents to develop and communicate family guidelines discouraging underage drinking. Peer leadership training was introduced in seventh grade. Community-level influences on underage drinking were gradually introduced by 8th grade, culminating in peer action teams during high school and a more complex curriculum in 11th grade. The name and con- tent of Project Northland programs changed annually to mark developmental changes in the cohort.
Social resistance skills training	An 8-week curriculum in seventh grade focused on developing skills to resist peer pressure as well as opportunities for peer leaders to plan alcohol-free activities until the students graduated from high school.
Normative education	Changes in norms concerning underage drinking were a major goal of the Project Northland interventions from 6th through 12th grades.
More broadly based skills training and comprehensive health education	Project Northland maintained a strong focus on alcohol but within that context taught youth leadership skills and ways to achieve developmental milestones of adolescence (e.g., autonomy and identity formation) without alcohol. Skills to identify and interpret unhealthy messages in the mass media also were taught.
Interactive teaching techniques	Peer leaders, role plays (including production of an improvisational theater piece in eighth grade), comics, fun games, alternative activities, and small-group projects were among the interactive teaching strategies used each year.
Teacher training and support	Part-time field staff were available at each intervention school. Teachers were given leave to attend half- or full-day training sessions before classroom implementation.
Adequate coverage and sufficient followup	Project Northland programs covered 6th through 12th grades and were successfully implemented with high participation rates each year.
Cultural sensitivity	People of color were represented in program materials, which included content specific to northern Minnesota Indian tribes and were sensitive to rural and small-town life in a northern climate. Programs were offered to a small school located on an Indian reservation outside the intervention districts when some of the cohort began transferring in and out of the study schools.
Additional components (e.g., family, community, and mass media initiatives)	Parent training and communitywide initiatives were part of Project Northland from sixth grade onward. Print media were used extensively throughout the program. (Radio and television could not be used because broadcasts could be received in reference communities.)
Evaluation	Project Northland was a randomized community trial using a cohort design beginning with sixth graders from 24 northern Minnesota school districts (*N* = 2,351; 91 percent of the eligible population). School districts were randomly assigned to either the intervention or the reference condition. Interventions were assessed using outcome measures that included an annual student survey; parent telephone interviews; observational studies of alcohol-purchase attempts by youthful buyers without age identification; and surveys of merchants, police, school principals, and community leaders.

1 The key components of effective programs were identified by Dusenbury and Falco (1995).

Although the Phase 1 interventions did not change the broader social environment (e.g., youth access to alcohol in the community) (Perry et al. 1996), the results suggest that Project Northland not only affected the targeted alcohol and other drug use behaviors but also influenced a number of their predictive factors, which are generally considered more resistant to change (Williams et al. in press). For example, after Phase 1, students in the intervention group scored significantly lower than the control group did on the Minnesota Multiphasic Personality Inventory–Adolescent (MMPI–A) Alcohol/Drug Problem Proneness Scale (Weed et al. 1994). The MMPI–A Proneness Scale measures risk factors for adolescent alcohol and other drug use, such as negative peer group influences, reduced involvement with parents, rule breaking, stimulus seeking, and lowered achievement orientation (Williams et al. in press).

## Challenges for Prevention During High School

**Figure 2:**
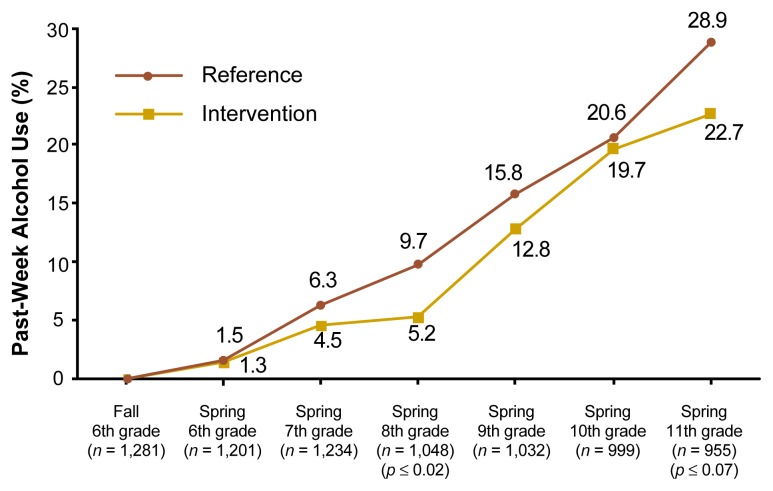
Past-week alcohol use rates across time for students who were nondrinkers at the start of Project Northland and who were present at the followup point indicated. ^1^Differences between the intervention and reference conditions were tested at each followup using mixed model regression methods (e.g., mixed model analyses of covariance). The unit of randomization (i.e., school district) was specified as a nested random effect.

The positive outcomes of Phase 1 of Project Northland have attenuated. This finding is consistent with other successful prevention programs (e.g., Bell et al. 1993) and is illustrated in figure 2, p. 112 with the rates of past-week alcohol use for students who reported no lifetime alcohol use at the sixth-grade baseline. During the interim period from 9th to 10th grade, when interventions were minimal, no statistically significant differences were found between students in the intervention and control communities on any of the alcohol use measures (although only past-week alcohol use is illustrated in figure 2). As the interventions resumed their level of intensity in 11th grade, the differences in past-week alcohol use for intervention and control group students who were nondrinkers at the start of the study approached significance. At the time this article was written, data collection had not been completed to determine program effects at the 12th-grade endpoint of the study. Figure 2 suggests that alcohol use during adolescence is a complex, ingrained social behavior, the prevention of which requires long-term interventions. Without such interventions, gains made during early adolescence may be lost.

Phase 2 of Project Northland included new strategies for the intervention students’ last years of high school that built on the interventions in early adolescence. Whereas Phase 1 emphasized strategies to encourage adolescents not to use alcohol, Phase 2 emphasized changing six community norms about adolescent alcohol use along with stimulating community action to reduce the availability of alcohol among high school students (Roski et al. 1997) (see textbox).

**Community-Level Norms for the High School Phase of Project Northland**

1. It is unacceptable for high school students to drink.
2. It is unacceptable for anyone (e.g., parent, older teen, merchant, or other adult) to provide alcohol to high school students.
3. Adults and high school students should take action when high school students are drinking.
4. Parents do have influence on their high school students’ drinking. Parents can provide social support, set clear expectations, monitor and supervise, and avoid inconsistent or excessively severe punishment.
5. Community events and public places are opportunities for modeling healthy behaviors for high school students.
6. High school students can have fun, establish their maturity and independence, and relieve stress and boredom without alcohol.

Although widespread acceptance existed during Phase 1 for the norm that drinking by sixth graders was undesirable (based on anecdotal and survey data from parents and students), no universal support was evident for norm 1, that drinking by high school students was unacceptable. In addition, we routinely heard that parents and other adults violated norm 2 (i.e., that it is unacceptable for anyone to provide alcohol to high school students) by providing alcohol for high school students’ parties or by purchasing alcohol for adolescents who could not purchase it for themselves. Norm 3 (i.e., that adults and high school students should take action when high school students are drinking) attempted to shift the focus away from the notion that underage drinking was an individual adolescent’s problem to the recognition that adults and high school students can intervene to reduce drinking during the high school years. Parents were encouraged (see prevention strategies below) to increase “protective factors” that reduced the likelihood that their child would drink (i.e., to provide social support, set clear expectations, monitor and supervise adolescents’ behavior, and avoid inconsistent or excessively severe punishment). Community members were educated (see prevention strategies below) to recognize that community events, such as summer festivals, and public places, such as parks, provided opportunities for adults to model healthy behaviors for high school students (norm 5). Norm 6 identified key issues of adolescence (i.e., to have fun, establish maturity and independence, and relieve stress and boredom) that advertisers and mass media often model as reasons for using alcohol. Those characteristics were successfully influenced in Phase 1 (i.e., young adolescents recognized that they did not need alcohol to have fun, establish their identity, and so forth). Phase 2 also addressed these issues, because we recognized that counter messages from the youth culture became more intense as the students progressed through high school.

## Five Prevention Strategies for High School

Phase 2 of Project Northland used five major strategies to reduce alcohol availability among youth and reinforce the norms listed in the textbox on p. 111. The strategies were community organizing, parent education, youth participation, media campaigns, and school curriculum. Seven local field staff were responsible for implementing the community organizing component in the 11 communities where the intervention students lived. The specific techniques used for community organizing, aimed at the outer ring in figure 1, included the following:

- One-on-one interviews with local citizens representing a broad spectrum of the community (averaging about 100 interviews per community)
- Formation of local action teams interested in implementing strategies to reduce underage access to alcohol in their geographical area and regional training sessions for team members on how to develop policy solutions to underage drinking in their geographical area
- Participation in community festivals (e.g., booths at fairs and distribution of petitions)
- Adoption of community policies (e.g., “gold card” programs, in which local businesses provided discounts to students who pledged to remain free of alcohol and other drugs and provide community service)
- Responsible beverage server (RBS) training sessions for retail outlets and bars (held by all communities to emphasize ways to reduce youth access)
- Compliance checks of age-of-sale laws (coordinated with local police or sheriffs’ departments).

Action teams initiated ordinances designed to reduce youth access to alcohol (e.g., youth curfews; mandatory RBS training; penalties to establishments for failure to check age identification; and noisy-assembly laws, which prohibit loud parties at certain hours), and three communities adopted at least one such ordinance during the 2 years of community organizing.

Parent programs have been a part of Project Northland since the first year. Therefore, it was a challenge during the last 2 years of the project to initiate new and developmentally appropriate programs for parents with considerable experience with the interventions. Some parents participated on action teams. In addition, short written materials were used to reach all parents of the intervention students. A postcard campaign of 11 cards, mailed at 6-week intervals, encouraged specific actions that parents could take to keep adolescents alcohol-free during high school (e.g., “The next time your teen goes to a party, call ahead to make sure there will be an adult chaperone.”). During the students’ senior year, materials were sent to all intervention parents and students encouraging them to communicate about alcohol by answering discussion questions and returning their answers to be eligible for a drawing of $500 per school. The discussion topics for the “Sound-OFF!” program included the minimum drinking age, adults providing alcohol to adolescents, and community responses to underage drinking. This program was widely publicized in the local media, and 20 percent of all intervention students and parents participated.

Youth participation has been a key component of Project Northland since its inception. Youth action teams were formed in 17 of the 18 intervention schools. Part-time, local, adult coordinators were hired to assist the teams, which met after school. Teams were brought together for two regional training sessions and to testify before the Minnesota legislature in support of a bill to reduce youth access to alcohol. These regional events underscored to the students that they were part of a larger, positive peer group in northeastern Minnesota that supported the Project Northland norms. The youth action teams not only targeted the first two rings of figure 1 but also worked for change in the broader social environment. More specifically, youth action teams were active in the following:

- Planning alcohol-free activities
- Planning safe homecoming, prom, or graduation activities
- Participating in community events, such as festivals and fairs
- Developing activities for “chemical health weeks” (e.g., mock crashes and “ghost-out” activities, in which a number of students would be identified as “victims” of alcohol-related crashes and act as ghosts throughout the school day)
- Decreasing underage access to alcohol by working to change local policies related to alcohol sales at community festivals and promoting family policies, such as providing safe and alcohol-free activities for youth in the home.

Several print-media campaigns during Phase 2 reinforced the efforts of the adult community organizing and the youth action teams by highlighting their activities. (The use of radio and television was not feasible, because those broadcasts also could be received in the reference communities.) Youth and adult community members were trained separately in media advocacy (e.g., writing and distributing press releases, contacting reporters, and pitching story ideas) to increase newspaper coverage of both the problems associated with underage alcohol use and solutions to those problems. Project staff developed media kits for each community and distributed Project Northland newsletters during the intervention period to highlight successes in the various communities. In addition, they distributed calendars to merchants licensed to sell alcohol in the intervention communities to assist clerks in calculating a buyer’s age. Over a 6-month period during the students’ senior year, project staff mailed monthly educational messages for insertion in bulletins to religious organizations in the intervention communities.

Although 18- to 20-year-olds cannot legally purchase alcohol themselves, they are likely to provide it to younger adolescents. Thus, a “Don’t Provide” campaign was developed with the assistance of an advertising agency to communicate to young adults (i.e., ages 18 to 22) that providing alcohol to high school students was unacceptable.^3^ The campaign included three posters for distribution to liquor and convenience stores, restaurants, bars, schools, and clinics; two corresponding postcards for the students’ parents; four postcards mailed to the target population of young adults; and mylar stickers for stores’ cooler doors. Messages were developed based on focus-group responses from young adults about what would be attention-getting and compelling. The messages included warnings about potential legal consequences (e.g., “Buy a minor a drink, and you could end up in the clink.”) as well as appeals to adolescents’ social responsibility and greater maturity (i.e., “Don’t provide alcohol to anyone under 21; they’re just kids for crying out loud.”). Local adult or youth action teams distributed the posters and stickers throughout the communities. Local newspapers provided campaign coverage, and one community developed a corresponding T-shirt.

The final media effort was a celebration and thank-you poster, entitled “Celebrating Success: How Project Northland Became a Prevention Model for the Nation,” which was mailed to approximately 5,500 community members (e.g., parents and students, police, merchants, community leaders, schools, and faith organizations). The poster included pictures of eight Project Northland volunteers, selected by nomination, to represent students, parents, police, school representatives, and members of the broader community.

During Phase 2 less reliance was placed on classroom curricula, which were the core of Phase 1 interventions. A classroom-based program was used only in 11th grade. That program involved a mock trial that emphasized the social and legal consequences of underage drinking. Although delivered within the classroom environment, the curriculum focused on the outer ring of the social environment in figure 1. It reinforced that underage drinking is a communitywide problem and gave students the opportunity to debate the legal intricacies of alcohol-related cases in six class sessions. The cases were developmentally appropriate and included the following:

- Personal injury or property damage resulting from the provision of alcohol to minors at home
- Use of alcohol by a pregnant adolescent
- Commercial alcohol sale to a minor and the resulting violence
- Drinking by minors at a community festival
- Lack of enforcement by a coach of the State athletic association’s rules against alcohol use
- Rape resulting from alcohol use at a party.

## Why Harm Reduction Is Not Used in Project Northland

Project Northland does not use a harm-reduction approach (e.g., Duncan et al. 1994; Milgram 1996). Harm reduction suggests that because alcohol and other drug use is prevalent during adolescence, a more effective prevention strategy would be to reduce the harm associated with use or promote “responsible use,” rather than emphasize “no use” prevention messages. However, alcohol use is illegal for all high school and most college students. Consequently, any harm-reduction approach would condone illegal behavior, which at best conveys a problematic message to adolescents. The reinstatement of the legal drinking age to 21 in all States not only reduced motor vehicle fatalities among 15- to 24-year-olds but also reduced other categories of violent deaths (Jones et al. 1992). Jones and colleagues (1992) reported three effects of raising the drinking age to 21: (1) delaying legal access to alcohol among adolescents, (2) preventing traumatic deaths that occur with legal access, and (3) delaying the onset of heavy drinking and associated fatal injuries. Therefore, maintaining the legal drinking age at 21 already appears to be an effective strategy for preventing alcohol problems among adolescents (O’Malley and Wagenaar 1991).

Recent prevalence data indicate that 45 percent of eighth graders in the United States have used alcohol in the past year, with 25 percent reporting past-month use (Johnston et al. 1996). Teaching responsible drinking skills is likely to be most effective before a person’s drinking behaviors become well established (i.e., at around ages 13 to 14 in the United States). Teaching the nuances of responsible drinking may be developmentally questionable for persons who are moving from concrete reasoning skills to more complex thinking. Furthermore, a study on brain maturation found evidence that five stages of maturation exist, with the last three occurring after age 10 (Hudspeth and Pribram 1992). Brain maturation stages seem to parallel the stages of cognitive development identified by Piaget through behavioral observations (Piaget and Inhelder 1966). The last brain maturation stage occurs late in adolescence (i.e., at ages 17 to 21) and is confined to further development of higher order reasoning. For example, understanding complex messages linked to desirable behaviors (e.g., “Although drinking is illegal for people your age, you can drink responsibly by limiting the amount you drink, drinking in homes with adult supervision, and avoiding driving by using a designated driver or calling for a ride.”) may not be possible for adolescents who have not progressed through the later stages of brain maturation and cognitive development. In addition, scientists do not know the effects of episodic heavy alcohol use, a typical drinking pattern of young people (Johnston et al. 1996), on brain maturation.

As discussed earlier in this article, research has indicated the following:

- Historical evidence exists that adolescence has not always been a time of heavy alcohol use (Johnson and Gerstein 1998).
- Significant morbidity and mortality are associated with underage drinking (Chassin and DeLucia 1996; NIAAA 1994).
- With earlier alcohol use, a significant increase occurs in the risk of developing an alcohol dependency or abuse disorder (Grant and Dawson 1997).

These findings, as well as others, suggest the need for consistent, simple messages advocating no alcohol use during adolescence. When delivered and reinforced in multiple ways, these messages may be the most effective alcohol prevention approach with the least amount of harm associated with it.

## Remaining Challenges for the Prevention of Alcohol-Related Problems During Adolescence

Although findings are still unclear on whether Project Northland has achieved its long-term goals of reducing alcohol use and alcohol-related problems in its cohort of adolescents, the achievement of several short-term goals is apparent. For example, outcomes show that community members will work with researchers to implement extensive programs targeting adolescents’ social environment over an extended time period. In addition, considerable support can be organized to reinforce community norms regarding the unacceptability of underage drinking and the value of healthy alternatives to adolescent alcohol use. Community leaders, including merchants, city council members, school officials, and parents, can be mobilized to provide sustained and consistent “no use” messages to students throughout their high school years. Those messages can emphasize appropriate consequences, as well as workable alternatives for meeting adolescents’ needs, without resorting to more ambiguous messages about “responsible use.” The effect of community-level interventions can be evaluated and policy decisions made on the basis of empirical findings. As analyses are initiated on the outcomes of Project Northland, information will be forthcoming about the challenges involved in fostering change at the community level.

The findings from the early adolescent phase of Project Northland are consistent with those of other researchers who have demonstrated lower alcohol use rates using similar strategies based on social learning theory (Botvin and Botvin 1992; Dusenbury and Falco 1995; Hansen 1993). Unfortunately, those types of programs are not used widely in U.S. schools, even though information is available on the types of programs most likely to be effective (Dusenbury and Falco 1995; Tobler and Stratton 1997). Whether and how those programs can be implemented on a large scale, how much it would cost (and who would pay), and how the interventions can affect the smaller group of adolescents who have already initiated drinking by sixth grade are questions that remain.

One area that Project Northland has not fully addressed is the role that mass media and advertising play in defining a youth culture that directs American youth to accomplish developmental tasks, such as autonomy and identity formation, by using tobacco and alcohol. The Minnesota tobacco trial has finally allowed public access to formerly internal documents indicating how the tobacco industry effectively marketed cigarettes to youth while deflecting responsibility by attributing youth smoking to peer pressure, parents, and a youth culture that they helped create through pervasive and potent advertising and promotional activities (Perry 1998). Parallels in the alcohol industry are evident. In addition, alcohol advertising has been found to include images or themes at odds with former Surgeon General Koop’s 1989 recommendations that alcohol advertising not portray activities that are dangerous when combined with drinking (e.g., driving and being near water) or that feature celebrities who are particularly appealing to youth (Madden and Grube 1994). Although research has not clearly substantiated the potential influence of advertising on youth drinking, recent studies suggest that alcohol advertising may predispose young people to drink (Grube and Wallack 1994). Even modest viewing of popular music video stations or televised sports may result in substantial exposure to glamorized depictions of alcohol and tobacco use (DuRant et al. 1997; Madden and Grube 1994).

The pervasiveness of media messages about tobacco and alcohol, the potency of the messages for adolescent audiences, and the placement of the messages where youth will see them (e.g., on billboards near schools) need to be better understood as potential contributors to adolescent drinking, rates of which are growing while the rest of the population is decreasing its alcohol consumption (Musto 1996). Comprehensive community efforts that include interventions designed to reduce media and advertising targeting of youth are fruitful areas for further prevention research.

## Footnotes

1 Lifetime alcohol dependence and abuse were defined using standard criteria from the American Psychiatric Association’s (APA’s) *Diagnostic and Statistical Manual of Mental Disorders, Fourth Edition* (APA 1994).

2 Figure 1 has been modified from the version used by Perry in her expert testimony in the 1998 tobacco trial in Minnesota *(State of Minnesota and Blue Cross and Blue Shield of Minnesota v. Philip Morris, Inc.; R.J. Reynolds Tobacco Company; Brown & Williamson Tobacco Corporation et al.).*

3 The Martin Williams advertising firm provided pro bono work in developing the “Don’t Provide” campaign.
